# Dataset for computational and experimental buckling analysis of constant-stiffness and variable-stiffness composite cylinders

**DOI:** 10.1016/j.dib.2024.110416

**Published:** 2024-04-14

**Authors:** Reece L. Lincoln, Paul M. Weaver, Alberto Pirrera, Evangelos Zympeloudis, Rainer M.J. Groh

**Affiliations:** a Bristol Composites Institute, University of Bristol, BS8 1UP; b Unit 29 Brookgate, South Liberty Lane, Bristol, BS3 2UN

**Keywords:** Nonlinear, Shells, Finite element, Testing

## Abstract

This dataset encapsulates comprehensive information and experimental outcomes derived from the buckling test of variable-stiffness composite cylinders subjected to axial compression. It is the first dataset about the correlation between experimental and computational analysis for a Rapid-Tow Sheared composite cylinder, a recently developed advanced composite manufacturing technique.

The data gathered during the test contains: raw test data for force, end-compression and strain gauges; and digital image correlation. The data for finite element validation is for a quasi-isotropic shell and variable-stiffness rapid tow-sheared shell. The data also contain imperfection signatures from a coordinate-measurement machine (CMM) of both cylinders.

This compilation of documented data stands as a robust resource for future investigations, enabling comparative analyses, validation of theoretical models, and advancements in the domain of designing and testing composite structures, particularly those employing variable-stiffness manufacturing techniques.

Specifications Table

**Table:** 

Subject	Aerospace Engineering
Specific subject area	Axially-compressed thin-walled cylinders are ubiquitous within aerospace engineering but have well-documented sensitivity to imperfections. The sensitivity to imperfections leads analysts to ‘overdesign’ cylindrical shells to compensate for this sensitivity. Advanced composite manufacturing techniques could enable engineers to circumvent this sensitivity to imperfections by placing continuous fibres (the load-bearing part of a composite structure) in a curvilinear path, tailoring the load path of a compressed structure. The first Rapid-Tow-Sheared (RTS) cylinder was designed, manufactured and tested at the University of Bristol in 2022. The RTS cylinder was compared to the current state-of-the-art constant-stiffness cylinder with a quasi-isotropic (QI) layup. The experiments and datasets represent a significant step towards lightweight, variable-stiffness composite shells being used in critical structures.
Data format	The dataset includes data that are raw and filtered.
Type of data	Data types include spreadsheets, software input files, csv files, images, scripts, graphs, and figures.
Data collection	Finite Element (FE) Analysis Finite element computations were performed using Abaqus/CAE 2018. The input files and results files are collected in the data. The manufactured imperfections of the cylinder were applied to the FE mesh of the cylinder through a Matlab 2021a live script (included in the data collection) and saved to the input file. End fixture rotations, as measured by the experimental data, were also applied to the cylinder in FE analyses and these data are captured in the sensitivity study. Force and displacement measurements A 500 kN hydraulically-operated Dartec machine was used to load and capture force and displacement data. Additionally, four linear variable differential transformers (LVDTs) were placed at 90 degree increments around the circumference to corroborate the Dartec data and capture rotations in the loading. Digital Image Correlation (DIC) A dotted pattern was applied onto a section of each cylinder to take surface deflection and strain measurements during the experimental tests using a two-camera DIC system. The location of DIC measurements was informed by FE predictions and the area of the DIC pattern on the cylinder was limited by practical applications (fitting a camera angle between the pillars of the testing machine). Strain gauges Strain gauges were also instrumented at nine and twelve locations across the QI and RTS cylinder, respectively, to complement and corroborate DIC measurements.
Data source location	The data were collected at the University of Bristol.
Data accessibility	Repository name: Data for: Manufacture and buckling test of a variable-stiffness, variable-thickness composite cylinder under axial compression Data identification number: 10.5523/bris.1zpa0tdgslky32c7htztoc03ee Direct URL to data: https://data.bris.ac.uk/data/dataset/1zpa0tdgslky32c7htztoc03ee Instructions for accessing these data: Open to download by following link
Related research article	Lincoln, Reece; Weaver, Paul; Pirrera, Alberto; Zympeloudis, Evangelos; Groh, Rainer. (2022). Manufacture and buckling test of a variable-stiffness, variable-thickness composite cylinder under axial compression. AIAA Journal 61(4):1849-1862. 10.2514/6.2022-0664.

## 1 Value of the Data

• These data represent the first axially-compressed Rapid Tow Sheared cylinder experimental data.
• These data represent the first imperfection signature from a manufactured Rapid Tow Sheard cylinder.
• These data are valuable to assess how finite element computational analyses can be matched with experimentation for axially-compressed cylinders.
• Researchers seeking to replicate experiments can see how the experimental data can be fed into a finite element simulation to get agreement between experiment and computation, and where discrepancies can occur.
• These data can be analysed with standard finite element packages.

## 2 Background

These data were compiled to accompany the publication [1] that describes the first manufactured and axially compressed Rapid Tow Sheared composite cylinder. The published article [1] describes the processes for experimentation and gives detail regarding the methodology and thought process behind the experiments and finite element correlations. The present data article adds value by giving greater detail to the published article and dataset and the relevance for researchers in the field of variable-stiffness cylinders. The current data article also gives greater detail on how the data are structured.

## 3 Data Description

The data are provided at the repository [2].

‘1 - Raw test data’ contains the data for both tests of the quasi-isotropic (QI) and Rapid Tow Sheared (RTS) cylinder. The spreadsheets contain raw and cleaned data for the force, displacement, LVDT, and strain gauge data. The ‘Raw data’ tab within each spreadsheet is the recorded data. The ‘Edited data’ tab within each spreadsheet contains: the time step, the axial strain (e.g., A11), the shear strain (e.g., A12), the hoop strain (e.g., A22), the LVDT data, and the Dartec displacement and force data. The strain gauge data (A through I) are references to the strain gauge locations in the ‘figs’ folder. The subsequent tabs are the axial, shear, hoop and Dartec data pulled out for individual plotting. The rotations, occurring naturally due to the slight eccentricity in loading, are calculated from the LVDT data.

‘2a – QI FE’ contains the data needed to perform FE analyses of the potted and unpotted QI cylinder. ‘2b – RTS FE’ contains the data needed to perform FE analyses of the potted and unpotted RTS cylinder. These data are to be run on Abaqus/CAE 2018. All that is primarily needed is the .inp file.

‘3 – CMM data and processing’ contains the imperfection data gathered from the CMM data for the QI and RTS cylinders. This folder also contains the Matlab 2021a Live Scripts necessary for finding a best-fit cylinder from these measurements for both the QI and RTS cylinders. Both QI and RTS spreadsheets have three columns that represent an (x, y, z) point cloud in space where the measurements were taken on the outside of the cylinder surface.

‘4 – DIC data’ contains digital image correlation data for a few select frames of the QI and RTS cylinder at pre- and post-buckling. Additionally, there is a Matlab 2021a Live Script that can process these data and show the post-buckled shape of the QI and RTS cylinders.

‘5 – SensitivityStudy’ contains Abaqus/CAE 2018 input files for various combinations of rotations applied to the RTS and QI cylinder as calculated from the LVDT data.

‘figs’ contains all fig. that act as references to locations and dimensions of the cylinder and experimental setup.

## 4 Experimental Design, Materials and Methods

### 4.1 Code

Code included to analyse the data includes Matlab 2021a live scripts for post processing the CMM data and DIC data.

### 4.2 Software

Software needed to analyse the data include: Microsoft Excel, Matlab 2021a, Abaqus/CAE 2018 and a text reader.

### 4.3 Experiment

**Fig. 1:**
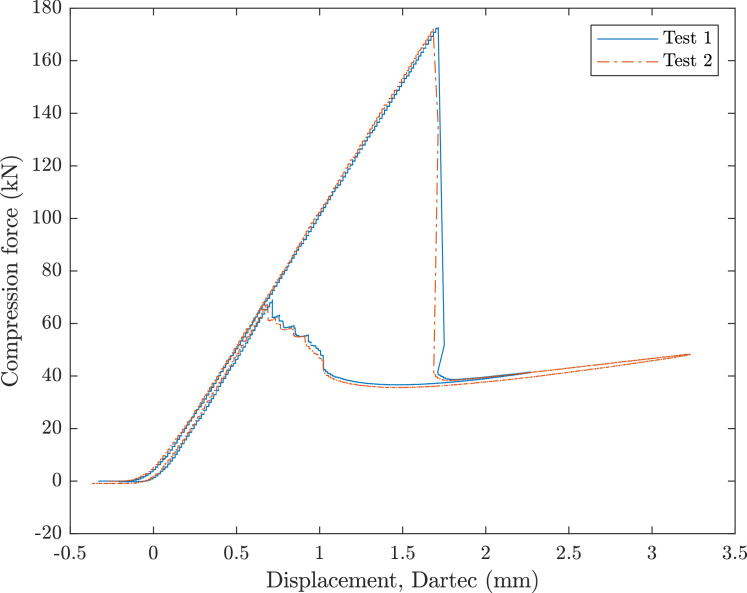
RTS load displacement data for two axial compression tests.

**Fig. 2:**
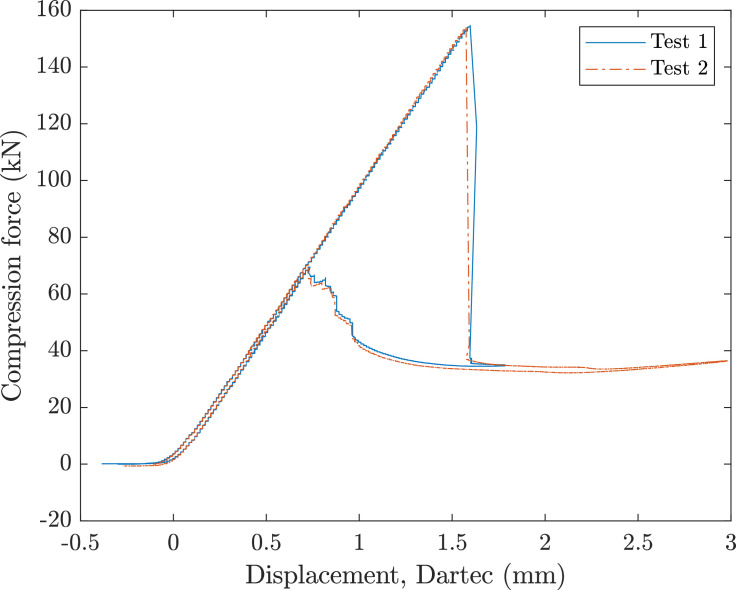
QI load displacement data for two axial compression tests.

**Fig. 3:**
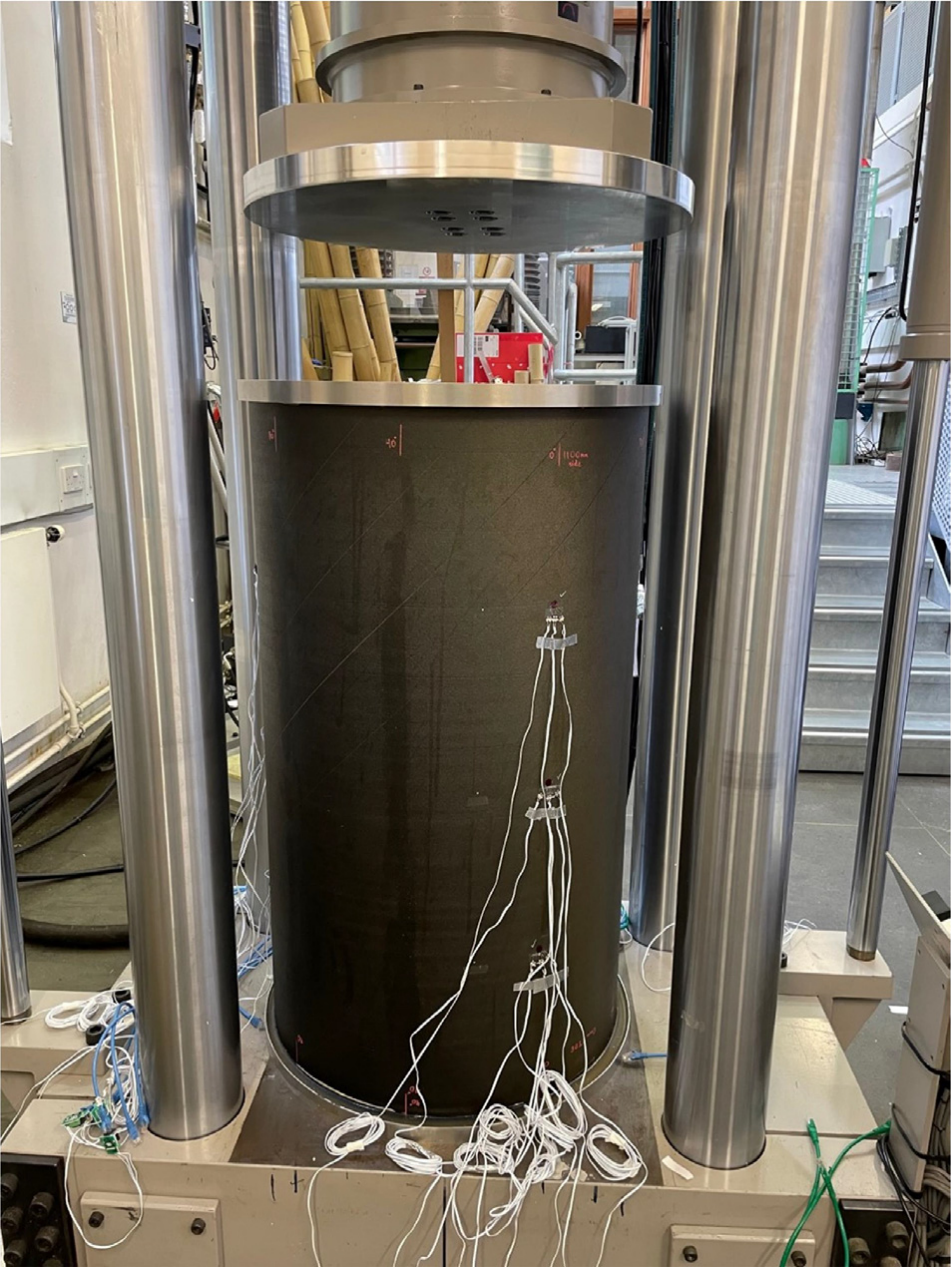
Experimental setup for compression of RTS cylinder. Note end-potting rings to distribute load.

A 500 kN Dartec test machine applied axial compression to the cylinders, clamped with end-potting. Fig. 1, Fig. 2 show the force and displacement data for the RTS and QI cylinder, respectively. A custom loading plate accommodated the ≈ 650 mm end-potting ring. Fig. 3 shows the experimental setup. Initial contact was confirmed by measuring nominal load and assessing parallelism with a feeler gauge (0.05-0.1 mm tolerance). Prebuckling stiffness aligned with predictions, allowing progression. Testing included an initial loading (up to 20% of predicted buckling displacement) with minor settling due to tolerances. Post-surface buckling inspections for visible material failure, followed by hysteresis analysis. A deep post-buckling state (3 mm end-shortening) was induced before unloading.

**Fig. 4:**
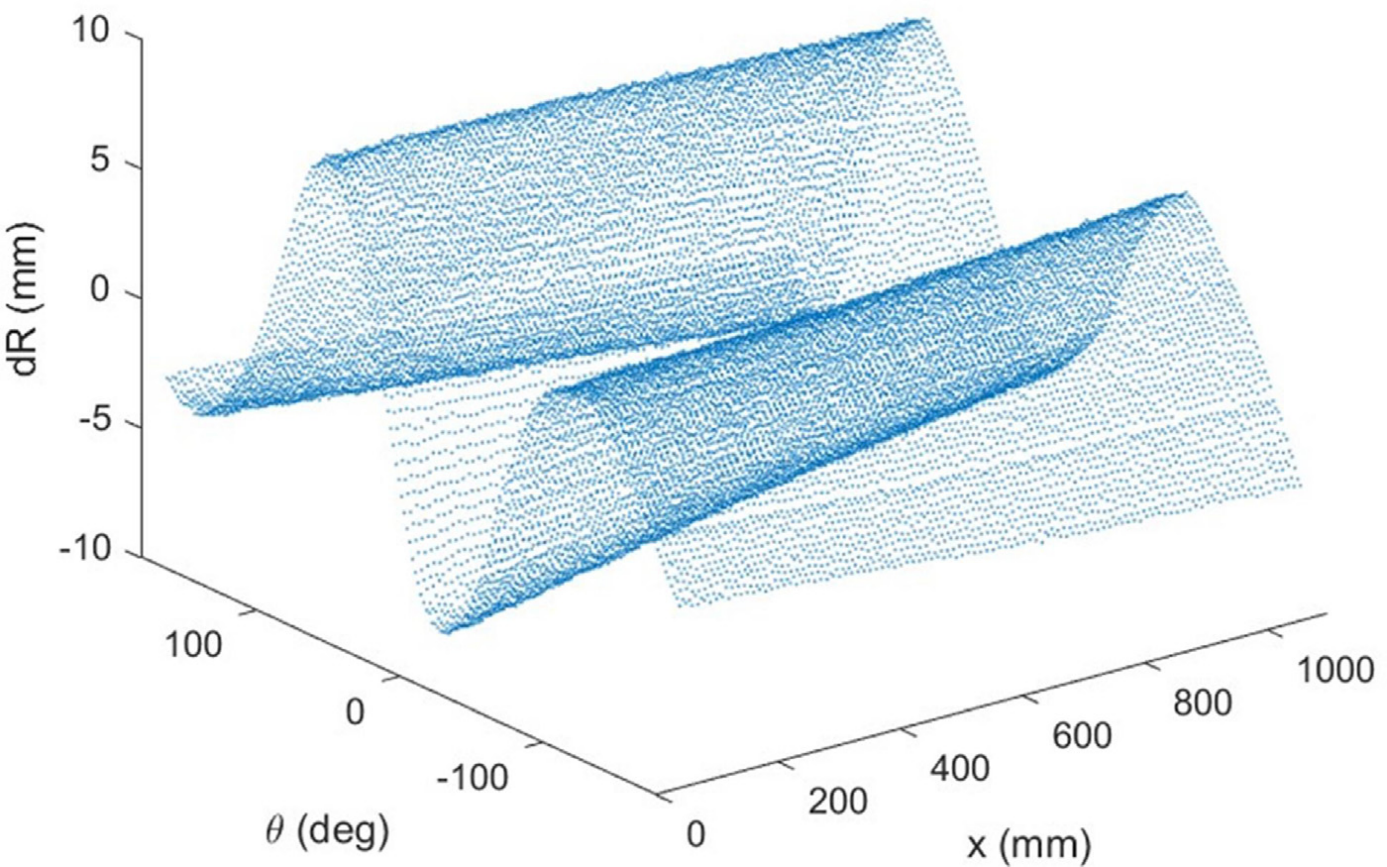
QI imperfection field with respect to the nominal inner radius of 300 mm. dR represents the radial deviation (outwards = positive, inwards = negative).

Geometric imperfections (the waviness of the surface of the cylinder) were measured before the experiment for both cylinders on a coordinate measurement machine (CMM). Several thousand points were probed with the CMM across the surface of the structure with respect to a nominal location. Fig. 4 shows the imperfection field as measured for the QI cylinder. This imperfection field was transformed into Fourier modes and used within the finite element analysis.

The rotational imperfections (eccentric loading) was measured with four Linear Variable Differential Transformers (LVDTs). The difference in displacements between the four LVDTs was used to reconstruct a rotation in two orthogonal radial directions. The calculated rotation was applied in finite element analyses.

The post-buckled shape of the cylinder was measured with a two-camera Digital Image Correlation (DIC) setup. These results were compared with finite element analyses.

### 4.4 Finite element analysis

**Fig. 5:**
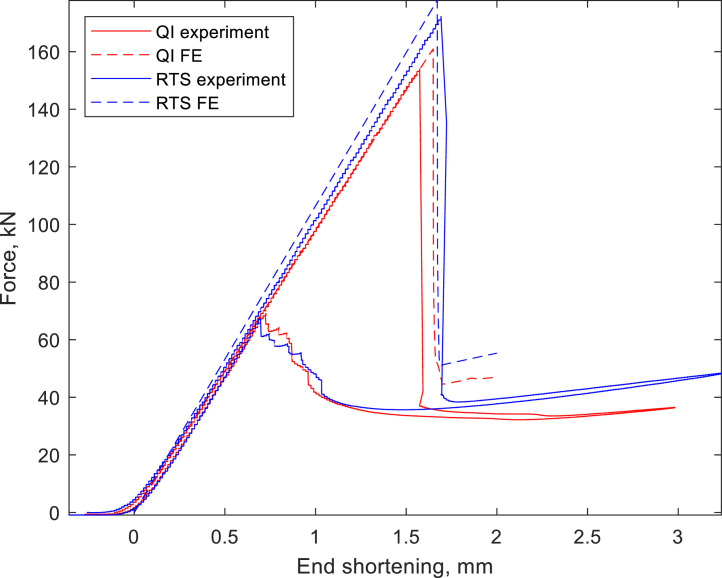
Experimental data vs FE data for QI and RTS cylinders. Experimental data is from test 1.

Nonlinear buckling load computation used Abaqus/CAE 2018 with S4R shell elements, utilizing 234 circumferential and 145 axial elements for RTS and QI cylinders, determined through prior mesh studies [3,4]. An ELASTIC ‘lamina’ type material model was used with Abaqus, where the key lamina-level properties are defined and a layup is applied to sections of the shell. Numerical stabilization was set at 1×10^−6^ for accuracy. Fig. 5 shows the correlation between experimental data and FE data.

**Fig. 6:**
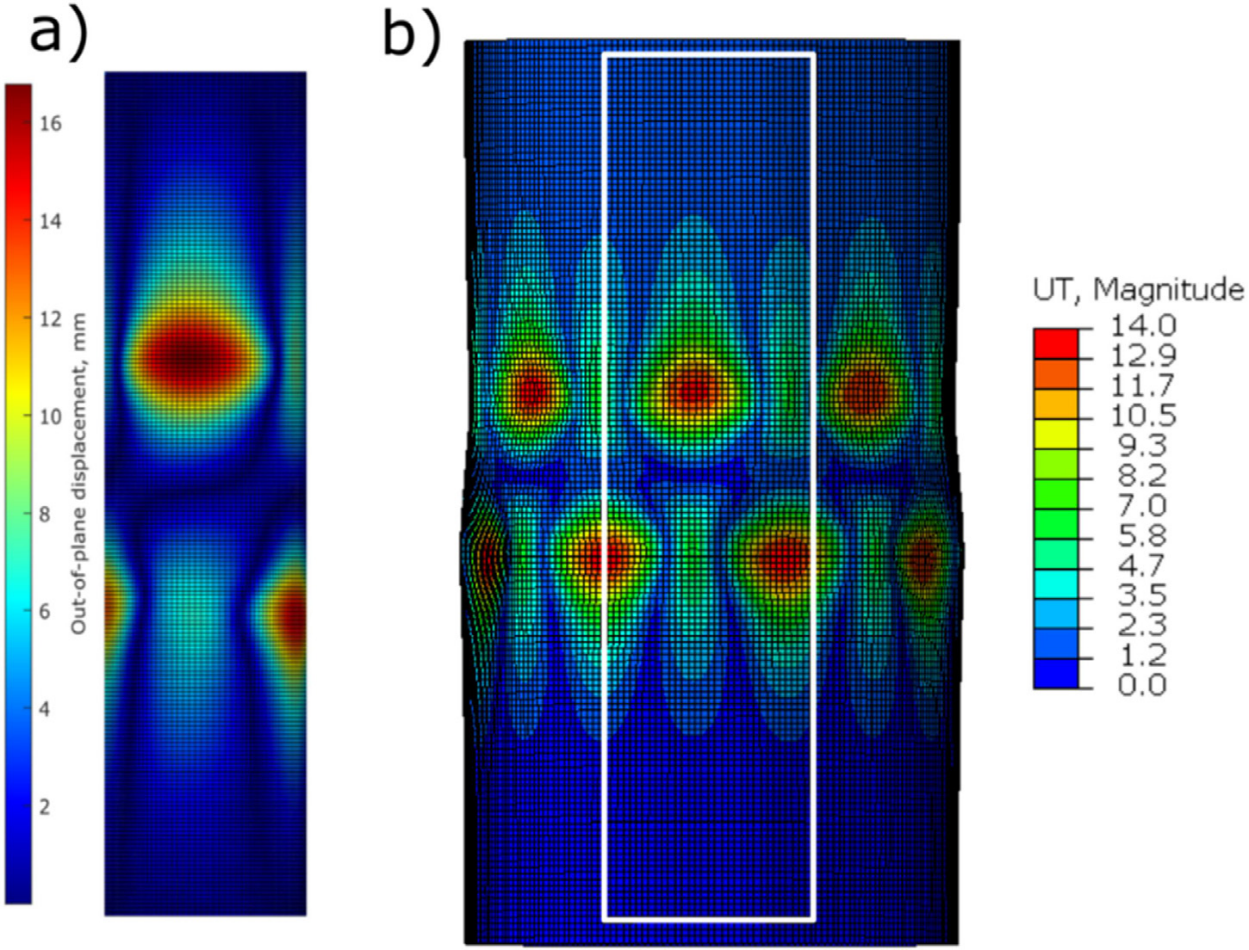
Comparison between a) experimental (DIC) post-buckled shape and b) numerical (Abaqus) post-buckled shape for the QI cylinder.

The geometric and rotational imperfections are applied through node manipulation and boundary conditions, respectively. A custom input file writer applied the geometric imperfections to the nodes with the Fourier modes that represent the imperfect surface. The rotational imperfections were applied as fixed rotational boundary conditions through the experiment. The post-buckled shape imperfections were determined in Abaqus and compared to experiment. These results can be seen in Fig. 6.

## Limitations

Some strain gauges during the experiments failed and therefore did not record data. Not all DIC data is included in the dataset as it would form too large of a dataset to upload.

## Ethical Statement

We confirm that the current work does not involve human subjects, animal subjects or any data collected from social media platforms. The authors have read and follow the ethical requirements for publication in Data in Brief.

## CRediT authorship contribution statement

**Reece L. Lincoln:** Conceptualization, Writing – original draft, Writing – review & editing. **Paul M. Weaver:** Supervision. **Alberto Pirrera:** Supervision. **Evangelos Zympeloudis:** Supervision. **Rainer M.J. Groh:** Supervision, Writing – review & editing.

## Data Availability

Data for: Manufacture and buckling test of a variable-stiffness, variable-thickness composite cylinder under axial compression (Original data) (data.bris).

## References

[1] Lincoln R.L., Weaver P.M., Pirrera A., Groh R.M.J., Zympeloudis E. (2023). Manufacture and buckling test of a variable-stiffness, variable-thickness composite cylinder under axial compression. AIAA J..

[2] A. Pirrera, R. M. Groh, and R. Lincoln, ‘Data for: manufacture and buckling test of a variable-stiffness, variable-thickness composite cylinder under axial compression’. University of Bristol, 2022. doi:10.5523/BRIS.1ZPA0TDGSLKY32C7HTZTOC03EE.

[3] Lincoln R., Weaver P., Pirrera A., Groh R.M. (2021). Optimisation of imperfection-insensitive continuous tow sheared rocket launch structures. AIAA Scitech 2021 Forum.

[4] Lincoln R.L., Weaver P.M., Pirrera A., Groh R.M.J. (2021). Imperfection-insensitive continuous tow-sheared cylinders. Compos. Struct..

